# The Effect of Acylcarnitines on Cognitive Function: A Two‐Sample Mendelian Randomization Study

**DOI:** 10.1002/brb3.70646

**Published:** 2025-07-03

**Authors:** Sisi Luan, Jinyan Zhang, Chenglong Wang, Ziqi Wang, Jianbo Zhou

**Affiliations:** ^1^ Department of Endocrinology, Beijing Tongren Hospital Capital Medical University Beijing China; ^2^ Beijing Tongren Hospital Capital Medical University Beijing China; ^3^ Plastic Surgery Hospital, Peking Union Medical College Chinese Academy of Medical Sciences Beijing China

**Keywords:** acylcarnitines, brain structure, cognition, dementia

## Abstract

**Objective:**

Clinical trials investigating the association between acylcarnitines and cognitive function have yielded conflicting results. We conducted a Mendelian randomization (MR) study to examine the causal associations between 20 acylcarnitines and neurocognitive outcomes.

**Methods:**

Single‐nucleotide polymorphisms of 20 acylcarnitines were extracted from primary European ancestry‐based genome‐wide association studies. Two‐sample MR (TSMR) was performed to initially screen for acylcarnitines potentially associated with cognitive performance. Further TSMR analysis was conducted to investigate the associations between specific acylcarnitines and other cognitive outcomes, dementia, and brain structure.

**Results:**

Among the 20 acylcarnitines, we observed that lower genetically predicted levels of butyrylcarnitine (*β* = −0.06, 95% CI: [−0.11 to −0.02], *p* = 0.003) and acetyl‐L‐carnitine (*β* = −0.02, 95% CI: [−0.04 to 0], *p* = 0.04) were associated with adverse neurocognitive effects. Furthermore, we found a negative correlation between low genetically predicted levels of butyrylcarnitine and cognitive function (*β* = −0.17, 95% CI: [−0.3 to −0.05], *p* = 0.01) as well as intelligence (*β* = −0.05, 95% CI: [−0.09 to −0.02], *p* = 0.003). However, there was no evidence supporting any association between these two acylcarnitines and dementia or brain structure.

**Conclusions:**

Our results suggest a causal association between acetyl‐L‐carnitine and butyrylcarnitine levels and adverse neurocognitive effects. Multicenter, multiregional, and large sample studies are needed to further validate these findings.

## Graphical abstract

This study is the first to employed TSMR to investigate the causal relationship between 20 acylcarnitines and neurocognitive outcomes. The findings indicate that lower genetically predicted levels of acetyl‐L‐carnitine and butyrylcarnitine are associated with diminished cognitive performance, underscoring their potential clinical implications in the early detection of cognitive decline.

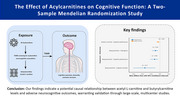

## 1 Introduction

Cognitive dysfunction is defined as deficits in one or more cognitive domains, including but not limited to memory, executive function, language, and spatial ability (Srikanth et al. 2020). It constitutes a core feature of many mental health disorders, including depression, schizophrenia, and Alzheimer's disease (AD) (Bolt et al. 2019; Dalili et al. 2015; Nikolin et al. 2021; Lyketsos et al. 2011). However, no medications have proven efficacious for addressing cognitive dysfunction (Langa and Levine 2014), making it an unmet clinical need. Therefore, there is a pressing demand to identify potential targets and supplements for the prevention and treatment of cognitive dysfunction.

Acylcarnitines are esters formed through the conjugation of fatty acids with L‐carnitine. The well‐established biological function of acylcarnitines is to facilitate the transport of acyl groups from the cytosol into the mitochondrial matrix for β‐oxidation, thereby generating energy to sustain cellular activity (Indiveri et al. 2011). Over the past few decades, additional neural actions and roles of acylcarnitines have been discovered, rendering them promising as therapeutic supplements. In older individuals, alterations in plasma levels of specific acylcarnitines have been proposed as predictive indicators for the progression to mild cognitive impairment, or AD (Mapstone et al. 2014; Cristofano et al. 2016). However, an increasing number of studies have presented rather heterogeneous results, casting uncertainty on the cognitive effects of specific acylcarnitines. In a study by Ciavardelli, patients with AD exhibited significantly lower plasma levels of several medium‐chain acylcarnitines (Ciavardelli et al. 2016), whereas Huguenard et al. reported higher levels of short‐chain acylcarnitines and lower levels of long‐chain acylcarnitines in AD (Huguenard et al. 2023). Meanwhile, although acetylcarnitine, the smallest acylcarnitine, is emerging as a supplemental option to alleviate the development of neurological disorders (Maldonado et al. 2020; McCann et al. 2021), its role in cognition remains contentious (Pennisi et al. 2020). In addition, the aforementioned studies investigating the relationship between acylcarnitines and cognition might be confounded by some unknown factors, complicating the assessment of causality. Mendelian randomization (MR) presents a causal inference methodology to examine the effect of modifiable exposure on a disease by leveraging genetic variants to provide evidence of robust associations. MR incorporates the advantage of summary statistics from large‐cohort genome‐wide association studies (GWASs), thereby minimizing residual confounding and reverse causation (Hemani et al. 2018). Recently, the assessment of genetic determinants of circulating metabolites has become feasible owing to the proliferation of large cohort studies employing both high‐throughput DNA sequencing data and untargeted metabolomic approaches.

To assess the potential causal relationship between acylcarnitines and cognition, two‐sample MR (TSMR) approaches were employed in this study. Data were obtained from a collection of complementary sources, including large‐scale GWAS pertaining to acylcarnitines (Shin et al. 2014), cognition (Mahedy et al. 2021; Lee et al. 2018; Savage et al. 2018; Davies et al. 2018), dementia (Chia et al. 2021; Kunkle et al. 2019), and brain structure (Hibar et al. 2015; Elliott et al. 2018).

## 2 Materials and Methods

### 2.1 Study Design

Our study adopted the TSMR design, an approach that leverages genetic variants as instrumental variables and utilizes summary statistics from two independent samples, which could minimize confounding and reverse causation and provide more robust evidence for causality. Another advantage of TSMR is its capacity for sensitivity analyses to identify unbalanced horizontal pleiotropy, a crucial step in meeting the assumptions of MR. We selected acylcarnitine as the exposure variable, while cognition, dementia, and brain structure served as the outcome variables. The data were primarily derived from the European Molecular Biology Laboratory‐European Bioinformatics Institute (EBI), within‐family GWAS, the Social Science Genetic Association Consortium (SSGAC), the FinnGen Project (FinnGen), the Alzheimer's Disease Genetics Consortium (ADGC), the International Parkinson Disease Genomics Consortium (IPDGC), and the Enhancing NeuroImaging Genetics Through Meta‐Analysis (ENIGMA) consortium (detailed in Table S1). To mitigate bias stemming from the overlap of exposure and outcome samples, we obtained exposure and outcome data from distinct databases. In selecting GWAS datasets for exposure and outcome variables, we considered factors such as the European ancestry of the population, a broad spectrum of disease types, larger sample sizes, and single nucleotide polymorphisms (SNPs). All the analyses were based on the requirements of the Strengthening the Reporting of Observational Studies in Epidemiology using the Mendelian Randomization (STROBE‐MR) checklist. This study relied on de‐identified summary‐level data that has been made publicly available, with ethical approval obtained in all original studies.

### 2.2 Acylcarnitine Genetic Instrument Selection and Data Source

**FIGURE 1:**
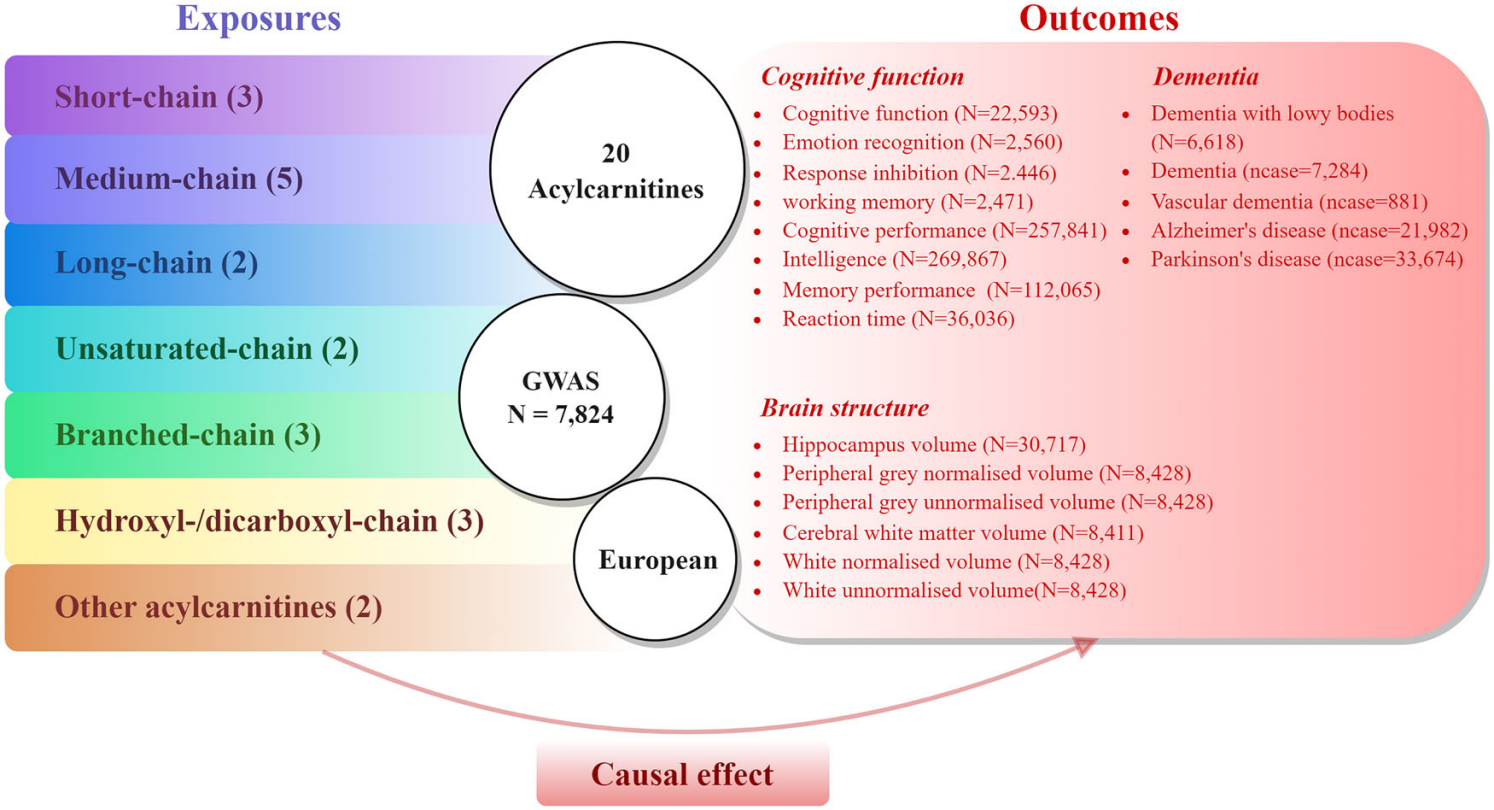
Study overview and Mendelian Randomization Model. All summary‐level genetic associations were derived from cohorts predominantly of European ancestry. Detailed information regarding the original genome‐wide association study (GWAS) for each drug‐target exposure and cognitive function endpoint included in the study is presented in Table **S1**. The causal effect pertains to the association of interest (the impact of exposure to decreased acylcarnitine levels on cognitive function, dementia, and brain structure). Single nucleotide polymorphisms (SNPs) were extracted from GWASs on acylcarnitines that surpassed the genome‐wide significance threshold (*p* < 5×10^−8^). The number in parentheses in the exposure section indicates the number of acylcarnitines within this classification. *N*: sample sizes; ncase: number of cases.

Exposures comprised 20 acylcarnitine obtained from a study on blood metabolites (*N* = 7824) conducted by Shin in 2014 (Shin et al. 2014) (Tables S2 and S3). The 20 acylcarnitine subtypes can be categorized into seven distinct types according to the carbon chain length and saturation level of the fatty acid moiety (Figure 1, Table S2). SNPs surpassing the generally accepted genome‐wide significance threshold (*p* < 5 × 10^−08^) for acylcarnitines were selected as instrumental variables. Subsequently, we clustered instrumental variables, considering the linkage disequilibrium (LD) structure derived from the 1,000 Genomes Project. Index SNPs (*R* ^2 ^< 0.01 with any other associated SNPs within 10 Mb) with the lowest *p*‐value were retained. A summary of each instrument is provided in Table S4.

### 2.3 Outcome Selection and Data Sources

#### 2.3.1 Cognition Outcomes

To comprehensively assess the effect of acylcarnitine on cognitive function, we examined eight cognitive function outcomes: cognitive performance from the SSGAC GWAS, which meta‐analyzed UK Biobank (UKB) fluid intelligence verbal‐numerical reasoning scores, alongside neuropsychological test data from the Cognitive Genomics Consortium (*N* = 257,841, standardized test score, mean = 0 ± 1) (Lee et al. 2018); cognition function data from the Within‐family GWAS meta‐analysis, encompassing at least three distinct cognitive scales (*N* = 22,593, standardized test score, mean = 0 ± 1); emotion recognition evaluated using the Emotion Recognition Task (*N* = 2560) (Mahedy et al. 2021); response inhibition gauged through the Stop‐Signal Task (*N* = 2446) (Mahedy et al. 2021); working memory assessed employing the N‐back Task (two‐back design, a continuous variable; *N* = 2471) (Mahedy et al. 2021); intelligence from a GWAS study conducted by Savage in 2018 (*N* = 269,867) (Savage et al. 2018); memory performance from the UKB, focusing on the frequency of errors in matching tasks (*N* = 112,065, number of errors on matching tasks, mean = 4.06 ± 3.23) (Davies et al. 2018); and reaction time from the UKB (*N* = 36,035, mean = 555.08 ± 112.19 ms) (Davies et al. 2018).

#### 2.3.2 Dementia Outcomes

We utilized five dementia‐related outcomes: dementia with Lewy bodies from a study conducted by Chia R (*N* = 6618) (Chia et al. 2021); AD from the International Genomics of Alzheimer's Project (IGAP), comprising four consortia: ADGC, Cohorts for Heart and Aging Research in Genomic Epidemiology Consortium (CHARGE), the European Alzheimer's Disease Initiative (EADI), and Genetic and Environmental Risk in AD/Defining Genetic, Polygenic and Environmental Risk for Alzheimer's Disease Consortium (GERAD/PERADES) (*N* = 21,982, onset of AD at or after 65 years of age) (Kunkle et al. 2019); Parkinson's disease from the IPDGC (*N* = 482,370); dementia data (*N* = 7284) and vascular dementia (*N* = 881) from FinnGen. These five datasets are binary datasets, and specific information regarding each dataset is provided in Table S1.

#### 2.3.3 Brain Structure Outcomes

We utilized data from six datasets encompassing six distinct brain regions: hippocampal volume from the ENIGMA Consortium GWAS meta‐analysis (*N* = 30,717) (Hibar et al. 2015); peripheral grey normalized volume, peripheral grey unnormalized volume, cerebral white matter volume, white normalized volume, and white unnormalized volume from a GWAS on brain imaging phenotypes conducted by Elliott LT in 2018 (*N* = 8,428) (Elliott et al. 2018).

### 2.4 Statistical Analysis and Interpretation of Results

To identify the specific acylcarnitine potentially significantly associated with cognitive function among the 20 acylcarnitines, we initially performed a TSMR analysis, employing cognitive performance as the outcome, to screen for associated acylcarnitines. F‐statistics (all F > 10, Table S4) indicated that the strength of selected genetic instruments was adequate. Subsequently, we standardized the selected acylcarnitine instrument exposures across all outcome datasets and performed correlated inverse‐variance weighted (IVW) MR (*n* ≥ 2) or Wald ratio (WR) MR (*n* = 1) as the primary analysis, and the relevant weighted median, simple mode, weighted mode, and MR‐Egger methods as auxiliary approaches. MR‐PRESSO was primarily used to detect and correct the outliers. This method was mainly used to remove SNPs that induced unanticipated variability during the analytical procedure. When a regression analysis was performed using the MR‒Egger model, heterogeneity was tested using the Cochran Q method. Standardized correlated MR effect estimates were computed per unit 1 log10 decrease in acylcarnitine levels. The analyses were performed using R software (version 4.3.1), leveraging the packages “TwoSampleMR”, “PRESSO”, “ggplot2”, and “MendelianRandomization” (Yavorska and Burgess 2017).

## 3 Results

### 3.1 Population Characteristics and Classification of Acylcarnitine

Acylcarnitine data were obtained from two population‐wide metabolite profiling endeavors, TwinsUK Resource (*N* = 6056) and KORA F4 (*N* = 7768). All summary‐level genetic associations were derived from cohorts predominantly comprising individuals of European descent (Table S3). Twenty acylcarnitines were categorized into seven groups, with specific characteristics of acetylcarnitine detailed in Figure 1 and Table S2.

### 3.2 Acylcarnitine Screening

To identify the specific acylcarnitine significantly associated with cognitive function among the 20 acylcarnitines, we initially performed TSMR analyses, employing cognitive performance as the outcome, with IVW and WR as primary models. We found that genetically predicted levels of butyrylcarnitine (*β* = −0.06, 95% CI: [−0.11 to −0.02], *p* = 0.003), acetyl‐L‐carnitine (β = −0.02, 95% CI: [−0.04 to 0], *p* = 0.04), and laurylcarnitine (*β* = −0.26, 95% CI: [−0.46 to −0.05], *p =* 0.01) were negatively associated with cognitive performance (Table S5). Given that laurylcarnitine exhibits only one SNP, the statistical power is limited. Thus, laurylcarnitine was not analyzed further.

### 3.3 Genetic Impact of Butyrylcarnitine on Outcomes

**FIGURE 2:**
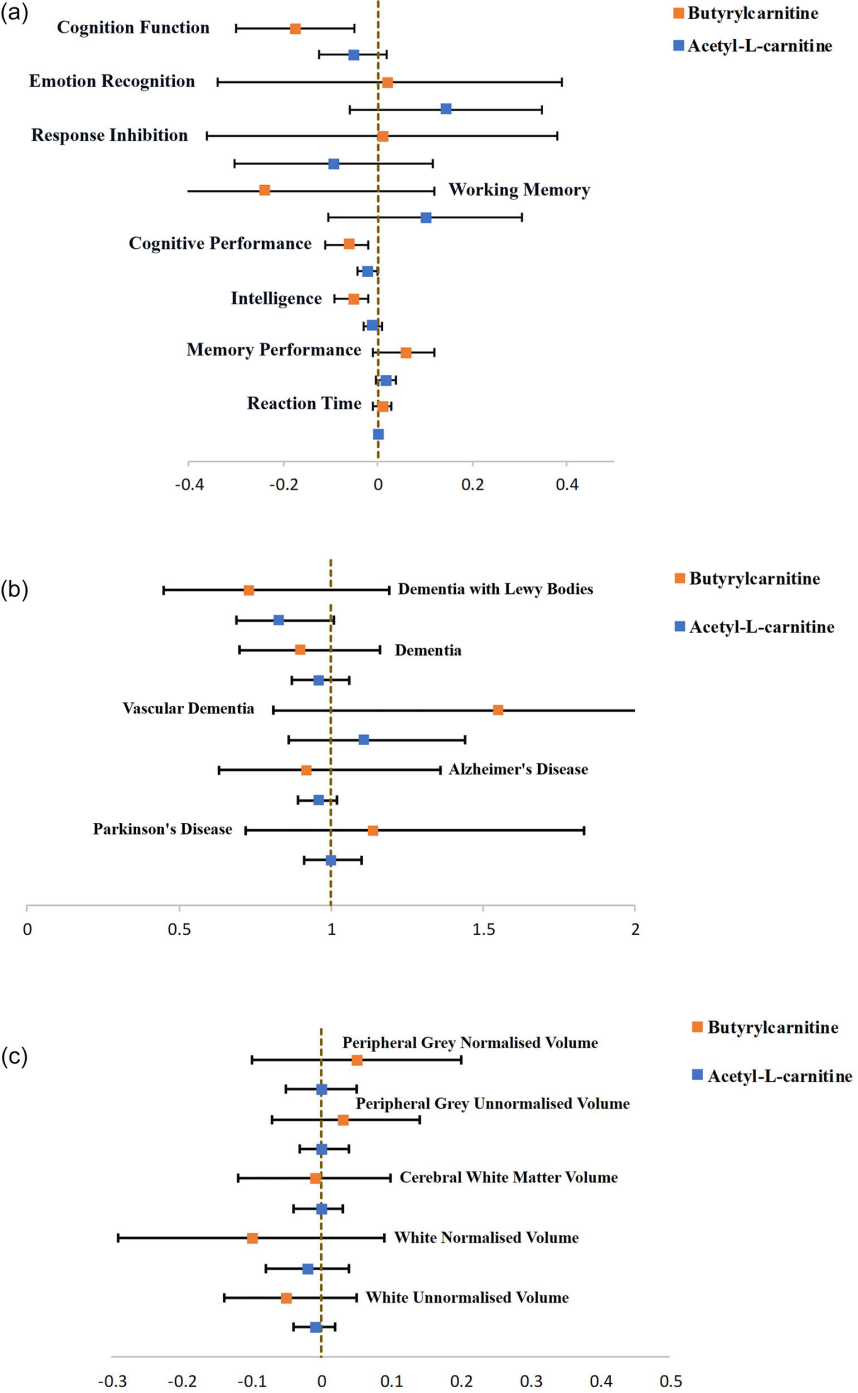
Genetic Impact of Decreased Butyrylcarnitine and Acetyl‐L‐carnitine Levels on Neurocognitive Outcomes. Butyrylcarnitine and acetyl‐L‐carnitine served as representatives of acylcarnitine exposure. Two‐sample Mendelian randomization (TSMR) was performed on neurocognitive phenotypes associated with general cognitive function, dementia, and brain structure. The data presented depicts standardized MR effect estimates and 95% confidence interval (CI) corresponding to 1 standard deviation (SD) decrease in exposures. (a) The genetic impact of decreased butyrylcarnitine and acetylcarnitine levels on cognition function. (b) The genetic impact of decreased butyrylcarnitine and acetylcarnitine levels on dementia. (c) The genetic impact of decreased butyrylcarnitine and acetylcarnitine levels on brain structure.

We conducted a series of TSMR analyses to further explore the relationship between butyrylcarnitine and cognition, dementia, and brain structure. The IVW model served as the primary model. We found significant causal effects between butyrylcarnitine and cognitive function (*β* = −0.17, 95% CI: [−0.3 to −0.05], *p* = 0.01) as well as intelligence (*β* = −0.05, 95% CI: [−0.09 to −0.02], *p =* 0.003) (Table 1; Figure 2a). The results suggested that lower genetically predicted levels of butyrylcarnitine are associated with adverse cognitive outcomes. However, no discernible causal link was observed between butyrylcarnitine and the manifestations of dementia or changes in brain structure (Table 1; Figure 2b,c). Furthermore, our analyses did not reveal any evidence of heterogeneity based on Cochran's Q test (*p* > 0.05) or pleiotropy according to MR‐Egger (*p* > 0.05) (Table S6).

**TABLE 1:** Potential Impact of Butyrylcarnitine on Cognitive Function, Dementia, and Brain Structure.

Exposure	Outcome	Unit	NSNPs	Beta (95%CI)	OR(95%CI)	*p*‐value
Butyrylcarnitine	**Cognitive Function**					
	Cognitive function	Standardized scores	4	−0.17 (−0.3 to −0.05)	0.84 (0.74 to 0.95)	0.01
	Emotion recognition	Standardized test scores	5	0.02 (−0.34 to 0.39)	1.02 (0.71 to 1.47)	0.91
	Response inhibition	Standardized test scores	5	0.01 (−0.36 to 0.38)	1.01 (0.7 to 1.47)	0.96
	Working memory	Standardized test scores	5	−0.24 (−0.61 to 0.12)	0.78 (0.54 to 1.13)	0.19
	Cognitive performance	Score 0 ± 1	5	−0.06 (−0.11 to −0.02)	0.94 (0.9 to 0.98)	< 0.01
	Intelligence	SD	4	−0.05 (−0.09 to −0.02)	0.95 (0.91 to 0.98)	< 0.01
	Memory performance	Score 4.06 ± 3.23	4	0.06 (−0.01 to 0.12)	1.06 (0.99 to 1.13)	0.07
	Reaction time	555.08 ± 112.19 ms	4	0.01 (−0.01 to 0.03)	1.01 (0.99 to 1.03)	0.36
	**Dementia**					
	Dementia with Lewy bodies	Binary	5	−0.31 (−0.8 to 0.17)	0.73 (0.45 to 1.19)	0.21
	Dementia	Binary	5	−0.1 (−0.35 to 0.15)	0.9 (0.7 to 1.16)	0.42
	Vascular dementia	Binary	5	0.44 (−0.21 to 1.09)	1.55 (0.81 to 2.98)	0.19
	Alzheimer's disease	Binary	3	−0.08 (−0.46 to 0.31)	0.92 (0.63 to 1.36)	0.69
	Parkinson's disease	Binary	4	0.13 (−0.33 to 0.6)	1.14 (0.72 to 1.83)	0.58
	**Brain structure**					
	Hippocampus volume	SD	5	−25.4(−87.22 to 36.43)	0 (0 to 6.7e+15)	0.42
	Peripheral grey normalized volume	SD	5	0.05(−0.1 to 0.2)	1.05 (0.91 to 1.22)	0.51
	Peripheral grey unnormalized volume	SD	5	0.03(−0.07 to 0.14)	1.03 (0.93 to 1.15)	0.51
	Cerebral White Matter volume	SD	5	−0.01(−0.12 to 0.1)	0.99(0.89 to 1.1)	0.83
	White normalized volume	SD	5	−0.1(−0.29 to 0.09)	0.91 (0.75 to 1.09)	0.3
	White unnormalized volume	SD	5	−0.05(−0.14 to 0.05)	0.95 (0.87 to 1.05)	0.33

Abbreviations: NA, not available; NSNPs, number of single nucleotide polymorphisms used as instruments in Mendelian randomization analysis.

**TABLE 2:** Potential Impact of Actyl‐L‐carnitine on cognitive function, dementia, and brain structure.

Exposure	Outcome	Unit	NSNPs	Beta (95%CI)	OR(95%CI)	*p*‐value
Acetyl‐L‐carnitine	**Cognitive Function**					
	Cognitive function	Standardized scores	2	−0.05 (−0.12 to 0.02)	0.95 (0.88 to 1.02)	0.15
	Emotion recognition	Standardized test scores	5	0.14 (−0.06 to 0.35)	1.16 (0.94 to 1.42)	0.16
	Response inhibition	Standardized test scores	5	−0.09 (−0.3 to 0.12)	0.91 (0.74 to 1.12)	0.39
	Working memory	Standardized test scores	5	0.1 (−0.1 to 0.31)	1.11 (0.9 to 1.36)	0.34
	Cognitive performance	Score 0 ± 1	5	−0.02 (−0.04 to 0)	0.98 (0.96 to 1)	0.04
	Intelligence	SD	4	−0.01 (−0.03 to 0.01)	0.99 (0.97 to 1.01)	0.31
	Memory performance	Score 4.06±3.23	4	0.02 (0 to 0.04)	1.02 (1 to 1.04)	0.1
	Reaction time	555.08±112.19 ms	4	0 (0 to 0.01)	1 (1 to 1.01)	0.62
	**Dementia**					
	Dementia with Lewy bodies	Binary	7	−0.18 (−0.37 to 0.01)	0.83 (0.69 to 1.01)	0.06
	Dementia	Binary	8	−0.04 (−0.14 to 0.05)	0.96 (0.87 to 1.06)	0.38
	Vascular dementia	Binary	8	0.1 (−0.16 to 0.36)	1.11 (0.86 to 1.44)	0.43
	Alzheimer's disease	Binary	5	−0.04 (−0.11 to 0.02)	0.96 (0.89 to 1.02)	0.21
	Parkinson's disease	Binary	7	0 (−0.09 to 0.1)	1 (0.91 to 1.1)	0.96
	**Brain structure**					
	Hippocampus volume	SD	5	−1.87 (−25.6 to 21.85)	0.15 (0 to 3.1e+09)	0.88
	Peripheral grey normalized volume	SD	8	0 (−0.05 to 0.05)	1 (0.96 to 1.06)	0.87
	Peripheral grey unnormalized volume	SD	8	0 (−0.03 to 0.04)	1 (0.97 to 1.04)	0.84
	Cerebral White Matter volume	SD	8	0 (−0.04 to 0.03)	1 (0.96 to 1.04)	0.82
	White normalized volume	SD	8	−0.02 (−0.08 to 0.04)	0.98 (0.92 to 1.04)	0.53
	White unnormalized volume	SD	8	−0.01 (−0.04 to 0.02)	0.99 (0.96 to 1.02)	0.49

Abbreviations: NA, not available; NSNPs, number of single nucleotide polymorphisms used as instruments in each drug‐target correlated Mendelian randomization analysis.

### 3.4 Genetic Impact of Acetyl‐L‐carnitine on Outcomes

We conducted a comprehensive TSMR analysis to delve deeper into the connection between acetyl‐L‐carnitine and cognitive function, dementia, and brain structure. The IVW model served as the principal analytical framework. While a notable causal effect was identified between acetyl‐L‐carnitine and cognitive performance, no such causal relationship was observed regarding other facets of cognition, dementia, or brain structures (Table 2; Figure 2). We found heterogeneity between acetyl‐L‐carnitine and cognitive performance (*p* < 0.01). Two SNPs, rs10466245 and rs12715455, exhibiting horizontal pleiotropy were excluded; rs10466245 was proximal to the *Marchf8* gene, and rs12715455 was proximal to the *Sfmbt1* gene. There was no obvious contradiction between the eliminated results and the original results. The functions of these genes are detailed in Table S7. In addition, no evidence of pleiotropy was detected according to MR‐Egger (*p* > 0.05) (Table S8).

## 4 Discussion

This study is the first to employ the MR method to investigate the potential causal relationship between circulating acylcarnitines and cognitive function. Our study indicates that reduced genetically predicted levels of acetyl‐L‐carnitine and butyrylcarnitine are associated with adverse neurocognitive outcomes, underscoring their potential clinical implications in the early detection of cognitive decline.

Acetyl‐L‐carnitine, an endogenous compound, not only plays a role in energy metabolism but also exhibits antioxidant properties. It protects against oxidative stress, modulates brain neurotransmitters, such as acetylcholine, serotonin, and dopamine, and traverses the blood–brain barrier to influence neurotrophic factors, such as nerve growth factor, and metabotropic glutamate receptors through epigenetic mechanisms (Huguenard et al. 2023). Some data from in vivo and in vitro studies suggest the potential of acetyl‐L‐carnitine as a therapeutic agent for AD and other neurodegenerative diseases, especially given its endogenous nature (Pogacnik et al. 2020). Serum acetyl‐L‐carnitine levels have been found to decrease along the spectrum from healthy individuals to those with cognitive impairment and AD (Cristofano et al. 2016). However, early studies suggested a favorable impact of acetyl‐L‐carnitine on cognition and behavior in aging populations, with subsequent studies and systematic reviews having failed to corroborate these findings, especially among patients with dementia (Hudson and Tabet 2003). The heterogeneity of results in previous studies may be attributed to the small sample size and the utilization of different methodologies and assessments. Moreover, most of the studies considered combinations of acetyl‐L‐carnitine with other therapies rather than acetyl‐L‐carnitine alone. Employing a novel MR approach, we were able to simulate such studies by eliminating confounding variables and assessing patterns of genetic susceptibility to specific neurocognitive outcomes. Our research corroborates previous findings that serum acetyl‐L‐carnitine levels decrease as cognitive impairment worsens, suggesting its potential as a biomarker for early cognitive decline. Given the brain's abundance of free‐radical‐generating iron and other compounds, the central nervous system is particularly susceptible to oxidative stress (Ames and Liu 2004). The antioxidant properties of acetyl‐L‐carnitine may reduce the release of reactive oxygen species from decaying mitochondria and attenuate protein oxidation in the brain (Poon et al. 2006). The maintenance of redox balance within the central nervous system forestalls cognitive decline and promotes cognitive longevity. Recent randomized controlled trials have further validated the protective effects of acetyl‐L‐carnitine on cognitive function (Malaguarnera et al. 2022). Therefore, supplementation with acetyl‐l‐carnitine may possess preventive potential for high‐risk populations, especially during the early phases of cognitive decline.

Butyrylcarnitine, a lipid marker indicative of whole‐body fatty acid oxidation and energy metabolism, has previously been linked to obesity, nonalcoholic fatty liver disease, and heart failure (Butte et al. 2015; Kalhan et al. 2011; Cheng et al. 2015). However, research into the role of butyrylcarnitine in the nervous system remains limited. An integrated multi‐omics analysis of sleep‐disordered breathing (SDB) traits associated with cognitive diseases suggests that butyrylcarnitine levels are implicated in SDB‐mediated signaling pathways, potentially establishing a connection between butyrylcarnitine and cognitive function (Kurniansyah et al. 2023). Concurrently, the food metabolome communicates with the brain through the circulatory system and interacts with hippocampal neurogenesis, a form of brain plasticity implicated in cognition and the etiology of depression. In a longitudinal aging cohort study, the dietary‐related metabolite butyrylcarnitine was found to modulate hippocampal neuronal differentiation (Du Preez et al. 2022). Another prospective longitudinal study validated the association between changes in butyrylcarnitine levels and cognitive improvement following olanzapine monotherapy in patients with schizophrenia and highlighted the potential effect of acylcarnitine on cognitive function (Zhao et al. 2023). In our study, incorporating a range of biological and clinical correlates of cognitive function expanded the scope and generalizability of the neuroprotective profile of butyrylcarnitine. Further research is warranted to explore the possible mechanisms underlying this relationship.

In this MR analysis, we employed robust instrumental variables, mitigating the risk of weak instrument bias, and verified key assumptions, ensuring no primary instruments were associated with potential confounders. Nevertheless, our study is limited by several factors: (1) MR is not a perfect substitute for authentic, long‐term, randomized controlled trials of acylcarnitines. Additional studies are imperative to validate the cognitive effects of acetyl‐L‐carnitine and butyrylcarnitine. (2) MR can detect genetic associations with high statistical precision; however, its efficacy is contingent upon the quality and scope of the data from which it draws its conclusions, and our analysis was limited to individuals of European ancestry. (3) Due to limitations in the human blood metabolite database, we only examined 20 acylcarnitines. Further research is warranted to investigate additional subtypes of acylcarnitines. (4) Cognitive assessments, including those of memory performance, reaction time, emotion recognition, response inhibition, and working memory, exhibit poor‐to‐moderate test–retest reliability. Therefore, measurement error may mask potential associations by widening confidence intervals (Hedge et al. 2018). (5) MR elucidates the lifelong impact of acylcarnitines on cognition, offering insights into the lifetime risk of exposure. However, it cannot discern effects at specific ages. Further studies utilizing genetic variants associated with acylcarnitines at distinct ages (e.g., childhood, and adulthood) are imperative to address this temporal aspect.

## 5 Conclusion

This MR study uncovers a causal relationship between reduced genetically predicted levels of acetyl‐L‐carnitine and butyrylcarnitine and adverse neurocognitive outcomes. To substantiate these findings, multicenter and multiregional studies with larger sample sizes are warranted.

## Author Contributions

**Sisi Luan**: conceptualization, writing – review and editing, writing – original draft, funding acquisition. **Jinyan Zhang**: methodology, software, data curation. **Chenglong Wang**: formal analysis, writing – review and editing. **Ziqi Wang**: writing – review and editing. **Jianbo Zhou**: conceptualization, funding acquisition, project administration, supervision.

## Conflicts of Interest

The authors declare no conflicts of interest.

## Peer Review

The peer review history for this article is available at https://publons.com/publon/10.1002/brb3.70646

## Supporting information




**Supporting Tables**: brb370646‐sup‐0001‐SuppMat.docx

## Data Availability

The data that support the findings of this study are available in ieu open gwas project at https://gwas.mrcieu.ac.uk. These data were derived from the following resources available in the public domain: ‐ ieu open gwas project, https://gwas.mrcieu.ac.uk.
