# Supplementary material for: The Effect of Acylcarnitines on Cognitive Function: A Two‐Sample Mendelian Randomization Study
Source: Brain Behav. 2025 Jul 3;15(7):e70646. doi: 10.1002/brb3.70646 (PMC12224038; doi:10.1002/brb3.70646)
Supplement: Supplementary file 1 — Supporting Tables: brb370646‐sup‐0001‐SuppMat.docx [file BRB3-15-e70646-s001.docx]

**Supplemental Text**

**Table S-1 Details of the Dataset.**

**Table S-2 Classification of Acylcarnitine.**

**Table S-3 Baseline Characteristics of the Population.**

**Table S-4** **SNP Information pertaining to 20 Acylcarnitines.**

**Table S-5** **Causal Relationship between 20 Acylcarnitines and Cognitive Performance.**

**Table S-6 Sensitivity Analysis of the Causal Effects between Butyrylcarnitine and Outcomes.**

**Table S-7 SNP Information pertaining to Butyrylcarnitine and Acetyl-L-carnitine.**

**Table S-8** **Sensitivity Analysis of the Causal Effects between Acetyl-L-carnitine and Outcomes.**

**Table S-1 Details of the Dataset**

| **Phenotypes** | **Consortium** | **First Author (Year)** | **Sample Size** | **N Cases** | **N Controls** | **Population** | **MRC-IEU ID:** | **Unit** | **PMID** |
| --- | --- | --- | --- | --- | --- | --- | --- | --- | --- |
|  |  |  |  |  |  |  |  |  |  |
| Acylcarnitines | NA | Shin (2014)^1^ | 7,824 | NA | NA | European | met-a-(474-753) | Log10 units | 24816252 |
| **Cognitive Function** | | | | | | | | | |
| Cognitive function | Within family GWAS | Howe LJ (2022) | 22,593 | NA | NA | European | ieu-b-4838 | Standardized scores | NA |
| Emotion recognition | NA | Mahedy (2021)^2^ | 2,560 | NA | NA | European | NA | Standardized test scores | 32335969 |
| Response inhibition | NA | Mahedy (2021) | 2,446 | NA | NA | European | NA | Standardized test scores | 32335969 |
| Working memory | NA | Mahedy (2021) | 2,471 | NA | NA | European | NA | Standardized test scores | 32335969 |
| Cognitive performance | SSGAC | Lee (2018)^3^ | 257841 | NA | NA | European | ebi-a-GCST006572 | Standardized test scores | 30038396 |
| Intelligence | NA | Savage JE (2018)^4^ | 269,867 | NA | NA | European | ebi-a-GCST006250 | SD | 29942086 |
| Memory performance | NA | Davies (2018)^5^ | 112065 | NA | NA | European | NA | Score log-transformed +1 | 29844566 |
| Reaction time | NA | Davies (2018) | 330069 | NA | NA | European | NA | Log-transformed millisecond | 29844566 |
| **Dementia** | | | | | | | | | |
| Dementia with Lewy bodies | NA | Chia R (2021)^6^ | 6,618 | 2,591 | 4,027 | European | ebi-a-GCST90001390 | Binary | 33589841 |
| Dementia | FinnGen | FinnGen (2021) | 7,284 | 209,487 | 216,771 | European | finn-b-F5_DEMENTIA | Binary | NA |
| Vascular dementia | FinnGen | FinnGen (2021) | 881 | 211,508 | 212,389 | European | finn-b-F5_VASCDEM | Binary | NA |
| Alzheimer's disease | ADGC | Kunkle BW (2019)^7^ | 21,982 | 41,944 | 63,926 | European | ieu-b-2 | Binary | 30820047 |
| Parkinson's disease | IPDGC | Nalls MA (2019) | 482,730 | 33,674 | 449,056 | European | ieu-a-7 | Binary | NA |
| **Brain Structure** | | | | | | | | | |
| Hippocampus volume | ENIGMA | Hibar (2015)^8^ | 30,717 | NA | NA | European | ieu-a-1045 | mm3 | 25607358 |
| peripheral grey normalised volume | NA | Elliott LT (2018)^9^ | 8,428 | NA | NA | European | ubm-a-1 | Continuous | 30305740 |
| peripheral grey unnormalised volume | NA | Elliott LT (2018) | 8,428 | NA | NA | European | ubm-a-2 | Continuous | 30305740 |
| Cerebral White Matter volume | NA | Elliott LT (2018) | 8,411 | NA | NA | European | ubm-a-2702 | Continuous | 30305740 |
| white normalised volume | NA | Elliott LT (2018) | 8,428 | NA | NA | European | ubm-a-7 | Continuous | 30305740 |
| white unnormalised volume | NA | Elliott LT (2018) | 8,428 | NA | NA | European | ubm-a-8 | Continuous | 30305740 |

NA: not available, GWAS: Genome-wide association study, SSGAC: Social Science Genetic Association Consortium, FinnGen: FinnGen Research Project, ADGC: Alzheimer Disease Genetics Consortium, IPDGC: International Parkinson Disease Genomics Consortium, ENGIMA: ENGIMA Consortium.

**Reference**

1 So-Youn Shin, Eric B Fauman, et al An atlas of genetic influences on human blood metabolites. Epub 2014 May 11.

2 Liam Mahedy, Steph Suddell, et al Alcohol use and cognitive functioning in young adults: improving causal inference. 2021 Feb;116(2):292-302.

3 James J Lee, Robbee Wedow, et al Gene discovery and polygenic prediction from a genome-wide association study of educational attainment in 1.1 million individuals. 2018 Jul 23;50(8):1112-1121.

4 Jeanne E Savage, Philip R Jansen, et al Genome-wide association meta-analysis in 269,867 individuals identifies new genetic and functional links to intelligence. 2018 Jul;50(7):912-919.

5 Gail Davies, Max Lam, et al Study of 300,486 individuals identifies 148 independent genetic loci influencing general cognitive function. 2018 May 29;9(1):2098.

6 Ruth Chia, Marya S Sabir, et al Genome sequencing analysis identifies new loci associated with Lewy body dementia and provides insights into its genetic architecture. 2021 Mar;53(3):294-303.

7 Kunkle BW, Grenier-Boley B, Sims R, et al Genetic meta-analysis of diagnosed Alzheimer’s disease identifies new risk loci and implicates Aβ, tau, immunity and lipid processing. Nat Genet. 2019;51(3):414-430.

8 Derrek P Hibar, Jason L Stein, et al Common genetic variants influence human subcortical brain structures. 2015 Apr 9;520(7546):224-9.

9. Lloyd T Elliott, Kevin Sharp, et al Genome-wide association studies of brain imaging phenotypes in UK Biobank. 2018 Oct;562(7726):210-216.

**Table S-2 Classification of Acylcarnitine**

| **Category** | **Acylcarnitines** |
| --- | --- |
| Short-chain acylcarnitines | Actyl-L-carnitine |
|  | Propionylcarnitine |
|  | Butyrylcarnitine |
| Medium-chain acylcarnitines | Octanoylcarnitine |
|  | Nonanoylcarnitine |
|  | Decanoylcarnitine |
|  | Cis-4-decenoylcarnitine |
|  | Laurylcarnitine |
| Long-chain acylcarnitines | Palmitoylcarnitine |
|  | Stearoylcarnitine |
| Unsaturated-chain acylcarnitines | Hexanoylcarnitine |
|  | 2-tetradecenoyl carnitine |
| Branched-chain acylcarnitines | Isobutyrylcarnitine |
|  | Isovalerylcarnitine |
|  | 2-methylbutyroylcarnitine |
| Hydroxyl-/dicarboxyl-chain acylcarnitines | Succinylcarnitine |
|  | 3-dehydrocarnitine |
|  | Hydroxyisovaleroyl carnitine |
| Other acylcarnitines | Glutaroyl carnitine |
|  | Oleoylcarnitine |

**Table S-3 Baseline Characteristics of the Population**

|  | **Twins UK**  **(n=6,056)** | **KORA F4**  **(n=7,768)** |
| --- | --- | --- |
| Country of origin | UK | Germany |
| male | 0.71 (433/6,056) | 0.48 (858/1,768) |
| Age, yrs | 53.4±14.0 | 60.8±8.8 |
| Body mass index, kg/m^2^ | 26.1±4.9 | 28.2±4.8 |

Twins UK: The UK Adult Twin Registry, KORA: The Cooperative Health Research in the Region of Augsburg.

**Table S-4 SNP Information pertaining to 20 Acylcarnitines**

| **EXPOSURE** | **SNP** | **A1** | **A2** | **CHR** | **EAF** | **SE** | **PVALUE** | **BETA** | **F statistics** |
| --- | --- | --- | --- | --- | --- | --- | --- | --- | --- |
| 2-methylbutyroylcarnitine | rs662138 | G | C | 6 | 0.1688 | 0.0034 | 3.65E-08 | -0.0187 | 30.25 |
| 2-tetradecenoyl carnitine | rs274567 | T | C | 5 | 0.3704 | 0.0042 | 1.27E-09 | 0.0257 | 37.44274 |
| 3-dehydrocarnitine | rs273913 | C | T | 5 | 0.6178 | 0.0025 | 1.08E-25 | -0.0261 | 108.9936 |
|  | rs316019 | C | A | 6 | 0.9014 | 0.0041 | 2.06E-08 | 0.0229 | 31.19631 |
|  | rs2291429 | C | A | 10 | 0.2373 | 0.0026 | 8.69E-11 | -0.0168 | 41.75148 |
| Acetyl-L-carnitine | rs1169299 | C | T | 12 | 0.46 | 0.01 | 3.86E-10 | 0.06 | 36 |
|  | rs1171617 | T | G | 10 | 0.76 | 0.01 | 6.53E-372 | 0.43 | 1849 |
|  | rs274551 | C | T | 5 | 0.83 | 0.01 | 1.88E-48 | 0.18 | 324 |
|  | rs77010315 | C | A | 5 | 0.99 | 0.04 | 1.61E-39 | 0.58 | 210.25 |
|  | rs10466245 | G | A | 10 | 0.77 | 0.01 | 4.81E-17 | 0.09 | 81 |
|  | rs853358 | T | A | 6 | 0.21 | 0.01 | 1.48E-20 | 0.1 | 100 |
|  | rs12715455 | T | A | 3 | 0.36 | 0.01 | 1.24E-13 | 0.07 | 49 |
|  | rs111653425 | T | C | 17 | 0.01 | 0.04 | 2.31E-10 | 0.28 | 49 |
| Butyrylcarnitine | rs274567 | T | C | 5 | 0.3712 | 0.0041 | 6.23E-09 | 0.024 | 34.26532 |
|  | rs1171617 | T | G | 10 | 0.7771 | 0.0047 | 1.61E-14 | 0.0358 | 58.01901 |
|  | rs4556628 | C | T | 12 | 0.7047 | 0.0042 | 6.98E-58 | -0.0679 | 261.3611 |
|  | rs11065202 | C | T | 12 | 0.4178 | 0.004 | 1.00E-200 | 0.1297 | 1051.381 |
|  | rs2469211 | T | A | 15 | 0.1079 | 0.006 | 1.06E-08 | 0.0342 | 32.49 |
| Cis-4-decenoyl carnitine | rs11161521 | C | T | 1 | 0.3017 | 0.0036 | 1.22E-63 | -0.0608 | 285.2346 |
|  | rs8396 | C | T | 4 | 0.3039 | 0.0037 | 8.96E-13 | -0.0262 | 50.14171 |
| Decanoylcarnitine | rs7552404 | G | A | 1 | 0.3016 | 0.0041 | 3.32E-43 | -0.0563 | 188.5598 |
|  | rs8396 | C | T | 4 | 0.3031 | 0.0041 | 8.69E-38 | -0.0533 | 169 |
|  | rs2062541 | A | G | 16 | 0.6144 | 0.004 | 2.89E-08 | 0.0225 | 31.64063 |
| Glutaroyl carnitine | rs13375749 | C | T | 1 | 0.2199 | 0.0027 | 2.80E-28 | -0.0297 | 121 |
|  | rs715 | C | T | 2 | 0.2826 | 0.0026 | 3.50E-16 | -0.0215 | 68.38018 |
|  | rs1981524 | T | C | 5 | 0.197 | 0.0027 | 3.15E-09 | 0.016 | 35.1166 |
|  | rs17641971 | C | T | 8 | 0.3392 | 0.0026 | 3.35E-22 | 0.0252 | 93.94083 |
|  | rs2291449 | G | A | 15 | 0.086 | 0.0043 | 1.44E-10 | 0.0276 | 41.19849 |
|  | rs8056893 | A | C | 16 | 0.7336 | 0.0026 | 2.23E-30 | -0.0302 | 134.9172 |
|  | rs246234 | G | C | 16 | 0.7042 | 0.0026 | 2.56E-14 | 0.0199 | 58.58136 |
|  | rs8012 | G | A | 19 | 0.5586 | 0.0027 | 6.36E-45 | -0.0374 | 191.8738 |
| Hexanoylcarnitine | rs11161521 | C | T | 1 | 0.3017 | 0.0033 | 6.42E-100 | -0.0703 | 453.8191 |
|  | rs2070630 | C | G | 4 | 0.2948 | 0.0034 | 6.76E-10 | -0.021 | 38.14879 |
|  | rs272869 | G | A | 5 | 0.6294 | 0.0033 | 1.40E-11 | -0.0224 | 46.0753 |
|  | rs1171615 | T | C | 10 | 0.7778 | 0.0037 | 4.05E-11 | 0.0246 | 44.20453 |
| Hydroxyisovaleroyl carnitine | rs2270968 | G | T | 3 | 0.7366 | 0.0037 | 1.31E-16 | -0.0307 | 68.84514 |
|  | rs274570 | T | C | 5 | 0.2962 | 0.0037 | 1.78E-09 | 0.0224 | 36.65157 |
| Isobutyrylcarnitine | rs7746102 | C | T | 6 | 0.2371 | 0.0043 | 6.50E-12 | 0.0296 | 47.38561 |
|  | rs662138 | G | C | 6 | 0.168 | 0.0052 | 4.98E-49 | -0.0767 | 217.5625 |
|  | rs2404602 | G | A | 15 | 0.5602 | 0.0036 | 3.56E-13 | -0.0259 | 51.76003 |
| Isovalerylcarnitine | rs11950562 | C | A | 5 | 0.4769 | 0.0027 | 2.31E-41 | -0.0362 | 179.7586 |
|  | rs2291449 | G | A | 15 | 0.0865 | 0.0052 | 2.35E-08 | 0.0288 | 30.67456 |
|  | rs9635324 | G | A | 15 | 0.3925 | 0.0027 | 1.89E-35 | -0.0337 | 155.7874 |
| Laurylcarnitine | rs4690909 | C | G | 4 | 0.6902 | 0.0049 | 1.06E-09 | 0.0301 | 37.73469 |
| Octanoylcarnitine | rs7552404 | G | A | 1 | 0.3016 | 0.004 | 1.27E-77 | -0.0746 | 347.8225 |
|  | rs8396 | C | T | 4 | 0.3028 | 0.0041 | 1.38E-31 | -0.0478 | 135.9215 |
|  | rs924135 | T | A | 16 | 0.616 | 0.0034 | 1.09E-09 | 0.0209 | 37.78633 |
| Oleoylcarnitine | rs273914 | T | A | 5 | 0.6322 | 0.0035 | 3.04E-11 | -0.0232 | 43.93796 |
| Palmitoylcarnitine | rs273914 | T | A | 5 | 0.6309 | 0.0033 | 4.02E-10 | -0.0209 | 40.11111 |
| Propionylcarnitine | rs7727544 | T | C | 5 | 0.5553 | 0.0018 | 6.61E-16 | -0.0148 | 67.60494 |
|  | rs662138 | G | C | 6 | 0.1701 | 0.0027 | 3.15E-14 | -0.0206 | 58.21125 |
|  | rs12356193 | G | A | 10 | 0.164 | 0.0027 | 1.96E-33 | -0.0328 | 147.5775 |
|  | rs2715311 | A | G | 18 | 0.3884 | 0.0018 | 6.57E-09 | -0.0107 | 35.33642 |
| Stearoylcarnitine | rs272881 | A | G | 5 | 0.617 | 0.0033 | 1.11E-10 | -0.0214 | 42.05326 |
| Succinylcarnitine | rs6703518 | T | G | 1 | 0.4273 | 0.0019 | 5.85E-09 | -0.0108 | 32.31025 |
|  | rs17806888 | C | T | 3 | 0.1194 | 0.0034 | 7.15E-11 | 0.0224 | 43.40484 |
|  | rs2686513 | C | T | 7 | 0.448 | 0.002 | 3.80E-08 | 0.011 | 30.25 |
|  | rs10988217 | G | A | 9 | 0.6117 | 0.0019 | 1.36E-18 | 0.0164 | 74.50416 |
|  | rs1472631 | G | A | 15 | 0.5081 | 0.0018 | 1.85E-88 | -0.0366 | 413.4444 |
|  | rs924135 | T | A | 16 | 0.6172 | 0.0018 | 1.52E-19 | 0.0167 | 86.07716 |
|  | rs8060756 | A | G | 16 | 0.3703 | 0.0018 | 4.07E-08 | 0.0102 | 32.11111 |
| X-13431--nonanoylcarnitine | rs12566232 | C | A | 1 | 0.2905 | 0.005 | 5.69E-19 | 0.0446 | 79.5664 |
|  | rs3738934 | C | T | 2 | 0.3769 | 0.0043 | 1.21E-134 | 0.1056 | 603.1022 |

SNPs: Single-nucleotide polymorphisms, A1: effect allele, A2: other allele, CHR: chromosome, EAF: effect allele frequency, SE: standard error.

**Table S-5 Causal Relationship between 20 Acylcarnitines and Cognitive Performance**

| **Exposure** | **Outcome** | **NSNPs** | **Beta(95%CI)** | **OR(95%CI)** | ***p*-value** |
| --- | --- | --- | --- | --- | --- |
| 2-methylbutyroylcarnitine | CP | 1 | -0.08(-0.47 to 0.3) | 0.92(0.63 to 1.35) | 0.67 |
| 2-tetradecenoyl carnitine |  | 1 | -0.07(-0.3 to 0.15) | 0.93(0.74 to 1.16) | 0.52 |
| 3-dehydrocarnitine |  | 3 | 0.01(-0.54 to 0.55) | 1.01(0.58 to 1.74) | 0.98 |
| Butyrylcarnitine |  | 5 | -0.06(-0.11 to -0.02) | 0.94(0.9 to 0.98) | < 0.01 |
| Cis-4-decenoylcarnitine |  | 2 | -0.07(-0.31 to 0.17) | 0.93(0.73 to 1.19) | 0.57 |
| Decanoylcarnitine |  | 3 | -0.09(-0.2 to 0.01) | 0.91(0.82 to 1.01) | 0.09 |
| Glutaroyl carnitine |  | 8 | 0.02(-0.15 to 0.2) | 1.02(0.86 to 1.23) | 0.79 |
| Hexanoylcarnitine |  | 4 | -0.07(-0.2 to 0.07) | 0.93(0.82 to 1.07) | 0.32 |
| Hydroxyisovaleroyl carnitine |  | 2 | 0.05(-0.28 to 0.39) | 1.05(0.75 to 1.47) | 0.77 |
| Isobutyrylcarnitine |  | 3 | 0.03(-0.17 to 0.22) | 1.03(0.85 to 1.25) | 0.78 |
| Isovalerylcarnitine |  | 3 | 0.1(-0.03 to 0.22) | 1.1(0.97 to 1.25) | 0.12 |
| L-actylcarnitine |  | 3 | -0.02(-0.04 to 0) | 0.98(0.96 to 1) | 0.04 |
| Laurylcarnitine |  | 1 | -0.26(-0.46 to -0.05) | 0.77(0.63 to 0.95) | 0.01 |
| Octanoylcarnitine |  | 3 | -0.07(-0.18 to 0.05) | 0.93(0.83 to 1.05) | 0.24 |
| Oleoylcarnitine |  | 1 | -0.09(-0.34 to 0.16) | 0.91(0.71 to 1.17) | 0.48 |
| Palmitoylcarnitine |  | 1 | -0.1(-0.38 to 0.18) | 0.9(0.69 to 1.19) | 0.48 |
| Propionylcarnitine |  | 4 | -0.09(-0.31 to 0.14) | 0.92(0.73 to 1.15) | 0.45 |
| Stearoylcarnitine |  | 1 | -0.1(-0.37 to 0.17) | 0.9(0.69 to 1.18) | 0.46 |
| Succinylcarnitine |  | 7 | -0.06(-0.33 to 0.2) | 0.94(0.72 to 1.23) | 0.65 |
| Nonanoylcarnitine |  | 2 | -0.02(-0.11 to 0.07) | 0.98(0.89 to 1.07) | 0.64 |

SNPs: Single-nucleotide polymorphisms, CI: confidence interval, CP: cognitive performance.

**Table S-6 Sensitivity Analysis of the Causal Effects between Butyrylcarnitine and Outcomes**

| exposure | outcome | MR-PRESSO$Pvalue | egger$intercept | egger$Pvalue | MR Egger$Q | MR Egger$Q_pvalue | IVW$Q | IVW$Q_pvalue |
| --- | --- | --- | --- | --- | --- | --- | --- | --- |
| Butyrylcarnitine | Cognitive function | | | | | | | |
|  | Cognitive function | 0.501 | -0.012 | 0.323 | 0.266 | 0.875 | 1.954 | 0.582 |
|  | Emotion recognition | 0.532 | 0.019 | 0.515 | 3.236 | 0.356 | 3.819 | 0.430 |
|  | Response inhibition | 0.526 | -0.008 | 0.782 | 0.852 | 0.836 | 0.943 | 0.918 |
|  | Working memory | 0.526 | 0.026 | 0.382 | 1.378 | 0.710 | 2.422 | 0.658 |
|  | Cognitive performance | 0.538 | 0.000 | 0.971 | 5.248 | 0.155 | 5.251 | 0.263 |
|  | Intelligence | 0.602 | 0.001 | 0.848 | 1.729 | 0.421 | 1.776 | 0.619 |
|  | Memory performance | 0.515 | 0.000 | 0.901 | 0.211 | 0.899 | 0.231 | 0.972 |
|  | Reaction time | 0.532 | 0.001 | 0.930 | 3.603 | 0.165 | 3.621 | 0.305 |
|  | Dementia | | | | | | | |
|  | Dementia with lewy bodies | 0.532 | -0.001 | 0.980 | 1.945 | 0.584 | 1.945 | 0.746 |
|  | Dementia | 0.522 | 0.005 | 0.794 | 2.417 | 0.491 | 2.498 | 0.645 |
|  | Vascular dementia | 0.523 | 0.025 | 0.625 | 0.999 | 0.802 | 1.293 | 0.863 |
|  | Alzheimer's disease | 0.506 | -0.010 | 0.541 | 1.218 | 0.544 | 1.754 | 0.625 |
|  | parkinson's disease | 0.519 | 0.011 | 0.557 | 2.565 | 0.464 | 2.998 | 0.558 |
|  | Brain structure | | | | | | | |
|  | Hippocampus volume | 0.536 | 2.987 | 0.543 | 0.422 | 0.936 | 0.891 | 0.926 |
|  | peripheral grey normalised volume | 0.530 | 0.015 | 0.230 | 1.554 | 0.670 | 2.089 | 0.719 |
|  | peripheral grey unnormalised volume | 0.513 | 0.011 | 0.203 | 1.525 | 0.677 | 2.167 | 0.705 |
|  | Cerebral White Matter volume | 0.534 | 0.010 | 0.292 | 1.575 | 0.665 | 3.200 | 0.525 |
|  | white normalised volume | 0.512 | 0.020 | 0.224 | 1.362 | 0.714 | 3.697 | 0.449 |
|  | white unnormalised volume | 0.515 | 0.010 | 0.223 | 1.537 | 0.674 | 3.888 | 0.421 |

MR: mendelian randomization, PRESSO: Pleiotropy RESidual Sum and Outlier, IVW: Inverse-Variance Weighted.

**Table S-7 SNP Information pertaining to Butyrylcarnitine and Acetyl-L-carnitine**

| SNP | Gene | effect_allele | other_allele | Role of genes |
| --- | --- | --- | --- | --- |
| **Butyrylcarnitine** | | | | |
| rs274567 | SLC22A5 | T | C | Polyspecific organic cation transporters in the liver, kidney, intestine, and other organs are critical for elimination of many endogenous small organic cations as well as a wide array of drugs and environmental toxins. |
| rs1171617 | SLC16A9 | T | G | Predicted to enable monocarboxylic acid transmembrane transporter activity. |
| rs4556628 | OASL | C | T | Enables DNA binding activity and double-stranded RNA binding activity. |
| rs11065202 | CABP1 | C | T | Calcium binding proteins are an important component of calcium mediated cellular signal transduction. |
| rs2469211 | ETFA | T | A | ETFA participates in catalyzing the initial step of the mitochondrial fatty acid beta-oxidation. |
| **Acetyl-L-carnitine** | | | | |
| rs1169299 | HNF1A | C | T | The protein encoded by this gene is a transcription factor required for the expression of several liver-specific genes. |
| rs1171617 | SLC16A9 | T | G | idem |
| rs274551 | SLC22A5 | C | T | idem |
| rs77010315 | SLC36A2 | C | A | This gene encodes a pH-dependent proton-coupled amino acid transporter that belongs to the amino acid auxin permease 1 protein family. |
| rs10466245 | 8-Mar | G | A | MARCH enzymes add ubiquitin to target lysines in substrate proteins, thereby signaling their vesicular transport between membrane compartments. |
| rs853358 | CD83 | T | A | The protein encoded by this gene is a single-pass type I membrane protein and member of the immunoglobulin superfamily of receptors. The encoded protein may be involved in the regulation of antigen presentation. |
| rs12715455 | SFMBT1 | T | A | This gene shares high similarity with the Drosophila Scm (sex comb on midleg) gene. It encodes a protein which contains four malignant brain tumor repeat (mbt) domains and may be involved in antigen recognition. |
| rs111653425 | SLC47A1 | T | C | This gene is located within the Smith-Magenis syndrome region on chromosome 17. It encodes a protein of unknown function. |

SNPs: Single-nucleotide polymorphisms.

**Table S-8 Sensitivity Analysis of the Causal Effects between Acetyl-L-carnitine and Outcomes**

| exposure | outcome | MR-PRESSO$Pvalue | egger$intercept | egger$Pvalue | MR Egger$Q | MR Egger$Q_pvalue | IVW$Q | IVW$Q_pvalue |
| --- | --- | --- | --- | --- | --- | --- | --- | --- |
| Acetyl-L-carnitine | Cognitive function | | | | | | | |
|  | Cognitive function | 0.505 | 0.007 | 0.47 | 2.064 | 0.356 | 2.87 | 0.412 |
|  | Emotion recognition | 0.506 | -0.003 | 0.894 | 7.221 | 0.204 | 7.249 | 0.298 |
|  | Response inhibition | 0.807 | 0.007 | 0.716 | 2.634 | 0.621 | 2.787 | 0.732 |
|  | Working memory | 0.363 | 0.002 | 0.93 | 8.269 | 0.082 | 8.287 | 0.141 |
|  | Cognitive performance | 0.517 | -0.005 | 0.411 | 51.294 | 0.001 | 57.953 | 0.001 |
|  | Intelligence | 0.883 | 0.003 | 0.65 | 29.466 | 0.001 | 31.227 | 0.001 |
|  | Memory performance | 0.557 | -0.002 | 0.637 | 5.531 | 0.237 | 5.888 | 0.317 |
|  | Reaction time | 0.53 | -0.001 | 0.301 | 4.699 | 0.319 | 6.349 | 0.273 |
|  | Dementia | | | | | | | |
|  | Dementia with lewy bodies | 0.427 | -0.035 | 0.218 | 5.872 | 0.437 | 7.763 | 0.353 |
|  | Dementia | 0.626 | -0.003 | 0.816 | 5.77 | 0.449 | 5.829 | 0.559 |
|  | Vascular dementia | 0.523 | -0.043 | 0.261 | 1.27 | 0.973 | 2.808 | 0.902 |
|  | Alzheimer's disease | 0.89 | -0.036 | 0.831 | 5.429 | 0.066 | 5.588 | 0.133 |
|  | parkinson's disease | 0.343 | -0.021 | 0.161 | 5.01 | 0.414 | 7.708 | 0.26 |
|  | Brain structure | | | | | | | |
|  | Hippocampus volume | 0.504 | -2.917 | 0.476 | 1.769 | 0.621 | 2.428 | 0.657 |
|  | peripheral grey normalised volume | 0.567 | 0.001 | 0.804 | 6.716 | 0.347 | 6.791 | 0.45 |
|  | peripheral grey unnormalised volume | 0.519 | 0 | 0.936 | 6.743 | 0.345 | 6.751 | 0.455 |
|  | Cerebral White Matter volume | 0.511 | 0.002 | 0.671 | 8.045 | 0.234 | 8.31 | 0.305 |
|  | white normalised volume | 0.551 | 0.004 | 0.643 | 6.546 | 0.364 | 6.804 | 0.449 |
|  | white unnormalised volume | 0.522 | 0.002 | 0.663 | 6.171 | 0.404 | 6.386 | 0.495 |

MR: mendelian randomization, PRESSO: Pleiotropy RESidual Sum and Outlier, IVW: Inverse-Variance Weighted.
